# A Rare Case of Massive Left Atrial Myxoma Presenting as Syncope

**DOI:** 10.7759/cureus.41249

**Published:** 2023-07-01

**Authors:** Adam Kurnick, Umida Burkhanova, Adam Friedman, Sabu John, Inna Bukharovich

**Affiliations:** 1 Department of Medicine, State University of New York (SUNY) Downstate Health Sciences University, Brooklyn, USA; 2 Department of Cardiology, State University of New York (SUNY) Downstate Health Sciences University, Brooklyn, USA; 3 Department of Cardiology, Kings County Hospital, Brooklyn, USA

**Keywords:** atrial myxoma, transthoracic echocardiography, left atrial mass, tte, syncope, myxoma, left atrial myxoma

## Abstract

We report a rare case of a large left atrial myxoma that manifested as syncope in a patient who presented to the hospital following a syncopal episode. Our patient had a history of hypertension and anemia with reported two months of dyspnea on exertion. He was found to have a large left atrial myxoma. Atrial myxomas are the most common benign primary cardiac tumors. Patients may be asymptomatic or experience shortness of breath, palpitations, syncope, or sudden death. Cases of syncope caused by left atrial myxoma have been rarely documented. Our case report adds to the growing literature documenting this phenomenon. Larger observational studies are needed to properly define the incidence of left atrial myxoma causing syncope.

## Introduction

Primary cardiac tumors are rare in the general population, and most are found to be benign. The most common benign cardiac tumor is an atrial myxoma, which most often presents in the left atrium [1-4]. Rarely reported are cases of left atrial myxomas causing syncopal episodes. We report a case of a 63-year-old male who presented after a syncopal episode and had also reported two months of dyspnea on exertion. A computed tomography angiography of the chest was negative for suspected pulmonary embolism; however, it revealed a large left atrial mass. A transthoracic echocardiogram was then obtained, which showed a large left atrial myxoma with prolapse into the left ventricle during diastole and resultant occlusion of the mitral orifice. This report is a rare finding of left atrial myxoma as the cause of syncope and adds to the sparse literature documenting this phenomenon.

## Case presentation

A 63-year-old male with a past medical history of hypertension and anemia presented to our emergency department following a syncopal event. While walking up the stairs at his home, he suddenly felt short of breath, had blurry vision, mild diaphoresis, and subsequently synopsized. There was no associated chest pain, nausea, or vomiting. This was his first syncopal episode, and he had no risk factors for developing deep vein thrombosis or pulmonary embolism. Family members recount that he appeared to be slightly confused and disoriented following the episode. He has had infrequent visits with his primary care physician and has never been seen by a cardiologist. He did endorse three months of dyspnea on exertion as well as when he climbs three flights of stairs.

Upon presentation, he was afebrile, his blood pressure was 142/117 mmHg, his heart rate was 102 bpm, and he was saturating 98% on room air. Physical examination was unremarkable other than pale mucosa, tachycardia, and absence of edema and jugular venous distention. Laboratory results are summarized in Table 1 and are significant for anemia, which was at his baseline hemoglobin level, and elevated pro-B-type natriuretic peptide.

**Table 1:** Laboratory results on admission.

Test name	Result	Reference range	Units
White blood cells	8.22	4.50-10.90	K/uL
Hemoglobin	11.2 (low)	14.0-18.0	g/dL
Platelets	253	130-400	K/uL
Sodium	139	136-146	mmol/L
Potassium	4.3	3.5-5.0	mmol/L
Blood urea nitrogen	12.0	8.0-23.0	mg/dL
Creatinine	1.07	0.70-1.20	mg/dL
Troponin T	0.010	≤0.010	ng/mL
Pro-B-type natriuretic peptide	930 (high)	≤125	pg/mL
COVID-19 screen	Not detected	n/a	n/a

**Figure 1:**
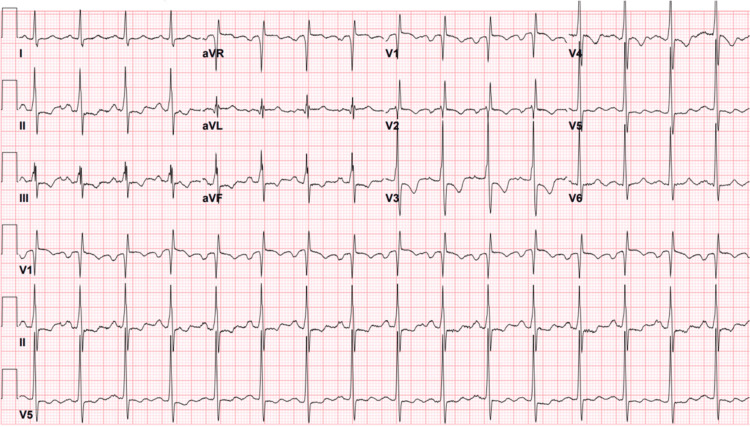
EKG on admission demonstrating normal sinus rhythm with non-specific ST-T wave changes.

**Figure 2:**
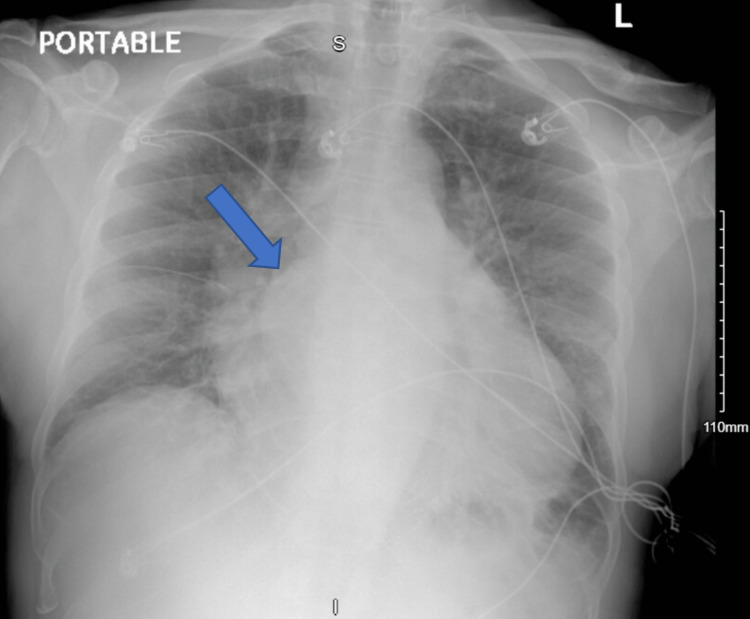
Chest X-ray revealing interstitial pulmonary edema and enlarged subcarinal opacity (arrow) suggesting right atrial enlargement.

**Figure 3:**
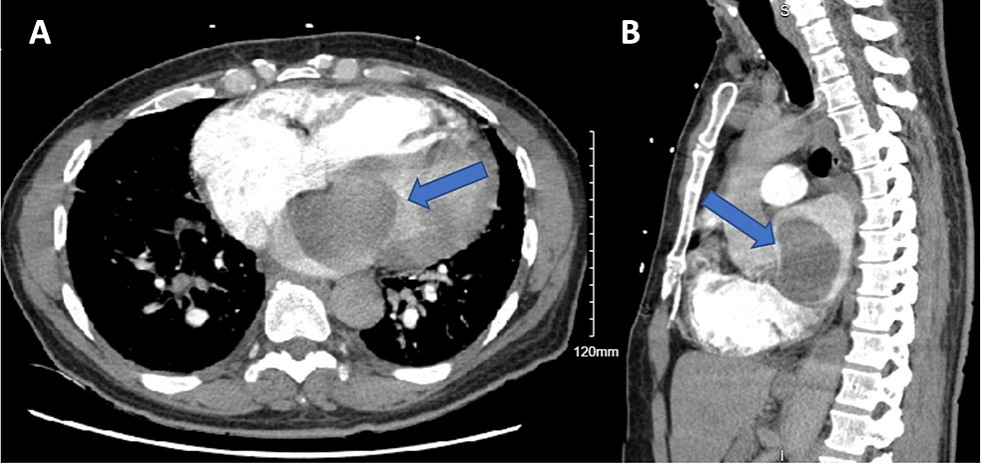
Computed tomography angiography of the chest. Image A shows an axial cross-section of the thorax at the level of the left atrium with a large filling defect reflecting the left atrial mass (arrow). Image B shows a sagittal cross-section with a left atrial filling defect representing mass (arrow).

**Figure 4:**
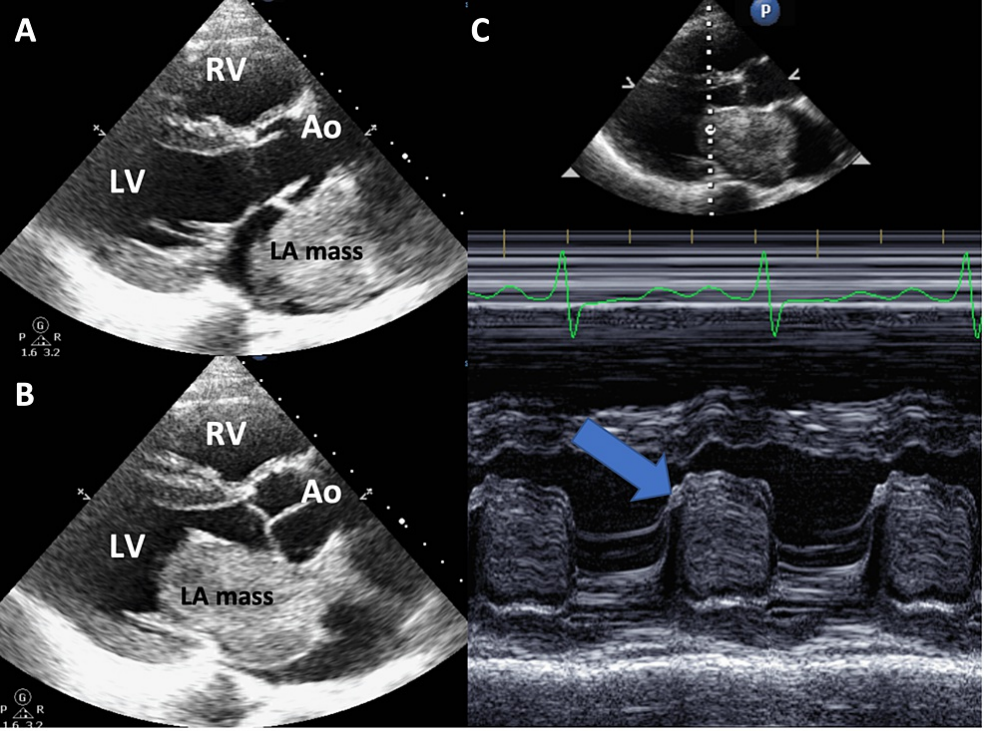
Transthoracic echocardiography in parasternal long axis view. Image A shows the large left atrial mass in LV systole. Image B shows the mass in the LV diastole protruding into the left ventricle, attached to the interatrial septum, with nearly complete obstruction of the mitral orifice. Image C is an M-mode view through the mitral leaflet showing the mass almost completely occluding the mitral orifice during LV diastole (arrow). LA, left atrium; LV, left ventricle; RV, right ventricle; Ao, aorta.

Electrocardiogram (EKG) was in normal sinus rhythm with non-specific ST-T wave changes (Figure 1). Chest X-ray revealed interstitial pulmonary edema in the setting of pulmonary vascular congestion. The cardiac silhouette was enlarged with a rounded subcarinal opacity suggesting right atrial enlargement (Figure 2). Computed tomography angiography (CTA) of the chest showed no pulmonary embolism but revealed a large well-marginated filling defect centered in the left atrium with extension across the mitral valve (Figure 3). There was also an enlargement of the pulmonary trunk measuring up to 3.5 cm, suggestive of pulmonary arterial hypertension. Transthoracic echocardiography (TTE) was completed and revealed a large mass in the left atrium attached to the interatrial septum, protruding through the mitral valve during left ventricle diastole, causing intermittent near-complete obstruction of the mitral orifice (Figure 4). The patient was evaluated by cardiothoracic surgery for a likely atrial myxoma.

## Discussion

Primary cardiac tumors are rare and are reported to be present in 0.001% to 0.3% by autopsy [1]. Of all primary cardiac tumors, cardiac myxomas are the most common and are most frequently found in the left atrium [2,3]. The prevalence of cardiac myxoma is approximately 0.03% in the general population, and the annual incidence is 0.5 per million individuals [4]. Primary benign cardiac tumors have an overall favorable prognosis [5]; however, embolization occurs in 30-40% of patients with myxomas and more frequently results in systemic embolization rather than pulmonary embolization given the prominence of left-sided myxomas over right-sided [6]. TTE is often the first imaging technique to establish a diagnosis of a cardiac tumor.

Clinical presentation varies and depends mainly on the size, positioning, and mobility of the myxoma. Approximately 3.2% to 46.4% of patients with cardiac myxoma are asymptomatic. Typically, symptoms occur when the myxoma prolapses through a valvular orifice and obstructs blood flow [7]. Symptoms include dyspnea, palpitations, syncope, tachycardia, and sudden death. Neurologic deficits may be seen in systemic embolization that reaches the brain. Surgical removal is the gold standard for treatment and should be done in haste given the risk of complications of embolization.

Our patient’s cardiac myxoma was first imaged by CTA of the chest, which was ordered to evaluate for pulmonary embolism given the patient’s history of syncope and dyspnea on exertion. Incidentally, a large left atrial mass was seen. The TTE showed a massive left atrial myxoma attached to the interatrial septum and nearly took up the entirety of space in the left atrium. Diastolic prolapsing of the mass into the left ventricle was seen with resulting obstruction of blood flow to the left ventricle and thereby the systemic circulation as well. The literature review revealed limited reports of left atrial myxoma causing syncope [8-12].

## Conclusions

In conclusion, cardiac tumors are rare findings that are most often benign and found to be cardiac myxoma. Cardiac myxomas are often first diagnosed on echocardiography and can be symptomatic based on the size, location, and mobility of the mass. Symptoms can include shortness of breath, palpitations, or sudden death. Instances of left atrial myxomas inducing syncope are rarely documented. We present a case of a large left atrial myxoma with associated extension into the left ventricle during left ventricular diastole causing a temporary mitral annular blockage. This case report adds to the sparse literature of reported cases of syncope caused by left atrial myxoma.
